# Enhancing cardiac pacing strategies: a review of conduction system pacing compared with right and biventricular pacing and their influence on myocardial function

**DOI:** 10.1093/ehjci/jeae090

**Published:** 2024-04-03

**Authors:** Mirakhmadjon Mirmaksudov, Stian Ross, Erik Kongsgård, Thor Edvardsen

**Affiliations:** Department of Cardiology, Oslo University Hospital, Rikshospitalet, Sognsvannsveien 20, 0372 Oslo, Norway; Department of Electrophysiology, Republican Specialized Scientific Practical Medical Center of Cardiology, Osiyo St. 4, 100052 Tashkent, Uzbekistan; Department of Cardiology, Oslo University Hospital, Rikshospitalet, Sognsvannsveien 20, 0372 Oslo, Norway; Department of Cardiology, Oslo University Hospital, Rikshospitalet, Sognsvannsveien 20, 0372 Oslo, Norway; Department of Cardiology, Oslo University Hospital, Rikshospitalet, Sognsvannsveien 20, 0372 Oslo, Norway; Institute of Clinical Medicine, University of Oslo, Sognsvannsveien 20, 0372 Oslo, Norway

**Keywords:** conduction system pacing, myocardial mechanics, left bundle branch area pacing, His bundle pacing, right ventricular pacing, biventricular pacing

## Abstract

Traditional right ventricular pacing (RVP) has been linked to the deterioration of both left ventricular diastolic and systolic function. This worsening often culminates in elevated rates of hospitalization due to heart failure, an increased risk of atrial fibrillation, and increased morbidity. While biventricular pacing (BVP) has demonstrated clinical and echocardiographic improvements in patients afflicted with heart failure and left bundle branch block, it has also encountered significant challenges such as a notable portion of non-responders and procedural failures attributed to anatomical complexities. In recent times, the interest has shifted towards conduction system pacing, initially, His bundle pacing, and more recently, left bundle branch area pacing, which are seen as promising alternatives to established methods. In contrast to other approaches, conduction system pacing offers the advantage of fostering more physiological and harmonized ventricular activation by directly stimulating the His–Purkinje network. This direct pacing results in a more synchronized systolic and diastolic function of the left ventricle compared with RVP and BVP. Of particular note is the capacity of conduction system pacing to yield a shorter QRS, conserve left ventricular ejection fraction, and reduce rates of mitral and tricuspid regurgitation when compared with RVP. The efficacy of conduction system pacing has also been found to have better clinical and echocardiographic improvement than BVP in patients requiring cardiac resynchronization. This review will delve into myocardial function in conduction system pacing compared with that in RVP and BVP.

## Graphical Abstract

**Graphical Abstract:**
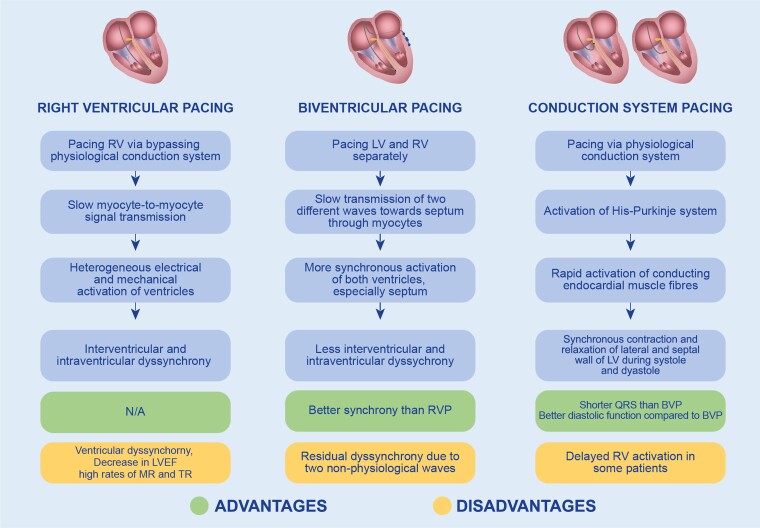

## Introduction

The conventional approach to pacing patients with bradyarrhythmias involves right ventricular pacing (RVP). However, this method has been associated with an elevated risk of heart failure–related hospitalizations and the development of pacing-induced cardiomyopathy, as indicated by various studies.^1^ It has been suggested that the abnormal electrical and mechanical activation patterns of the ventricles resulting from RVP, commonly referred to as ventricular dyssynchrony, is the main reason for pacing-induced cardiomyopathy.^2^ In contrast, biventricular pacing (BVP) has been designed to limit dyssynchrony, yielding benefits such as reverse remodelling, enhanced exercise tolerance, reduced hospitalization rates and mortality in patients with systolic heart failure, and a wide QRS with left bundle branch block (LBBB) morphology.^3^ BVP has also been proven effective for treating patients with pacing-induced cardiomyopathy post-RVP.^4^ However, this approach suffers from limitations by cardiac venous anatomy, phrenic nerve stimulation, and a notable rate of non-responders.^5,6^ Various clinical trials have revealed that 20–40% of patients fail to respond to cardiac resynchronization therapy (CRT) through BVP.^7^

Conduction system pacing has recently emerged as a novel pacing method, wherein a pacemaker lead is implanted close to the cardiac conduction system. Conduction system pacing encompasses His bundle pacing (HBP) and left bundle branch area pacing (LBBAP), offering a physiological alternative to BVP and RVP. A major advantage of conduction system pacing lies in its ability to nearly recover cardiac electrical depolarization and repolarization in patients with intrinsic conduction abnormalities.^8^ Furthermore, conduction system pacing exhibits superiority over BVP in terms of an incidence of sustained and non-sustained ventricular arrhythmias and new onset of atrial fibrillation.^9^

These various pacing methods elicit distinct myocardial activation patterns. RVP causes regionally different contractions, characterized by an early activation of the pacing site with a subsequent contraction of weaker myocardial regions, followed by a stronger contractile work of later-activated regions due to increased afterload.^10^ RVP can induce both systolic and diastolic dyssynchrony, as evidenced by an increased dispersion of strain values throughout the cardiac cycle.^11^ Acute RVP-induced dyssynchrony has been shown to adversely affect left ventricular (LV) longitudinal function and torsional deformation, as observed by speckle-tracking echocardiography.^12^ Another marker of LV dyssynchrony, septal flash, has been observed in the majority of patients undergoing conventional RVP, with its magnitude correlating with LV dysfunction and adverse remodelling.^13^ Alternative RVP strategies have been developed in order to reduce the negative impacts associated with traditional apical pacing. The most widely used strategies have been right ventricular (RV) septal and outflow tract pacing. Some clinical studies suggest that these alternative methods may improve LV synchrony. However, the results of such studies have been heterogeneous.^14,15^

While BVP mitigates dyssynchrony more effectively compared with RVP, it still causes a certain degree of residual dyssynchrony due to the merging of two non-physiological wavefronts.^16^ However, it has been found that the degree of dyssynchrony measured by time-to-peak shortening, strain patterns, or internal stretch fraction in conduction system pacing is moderate and nearly similar to that in physiological conduction and less than that observed in RVP.^17^

Computational remodelling studies have demonstrated that LBBAP predominantly restores LV function but may lead to increased RV overload.^18^ While several meta-analyses have reported improved LV ejection fraction (LVEF) with conduction system pacing compared with BVP, only limited research has delved into the field of myocardial dyssynchrony in patients undergoing conduction system pacing compared with BVP.^19,20^

This article serves to provide an in-depth review examining the impacts of conduction system pacing on myocardial function and clinical outcomes in comparison with RVP and BVP. Through this exploration, our aim is to illuminate the potential advantages of conduction system pacing in the realm of cardiac pacing strategies. Most papers with available echocardiographic data have focused on LVEF, QRS duration, and other traditional measurements of myocardial mechanics. We have thoroughly discussed these findings in the article. Furthermore, we have included more sophisticated approaches to evaluating LV function that may be present in the literature. Our review aims to provide a comprehensive overview, encompassing both established metrics and emerging methodologies in assessing myocardial function in the context of conduction system pacing.

## Outline of conduction system pacing (HBP and LBBAP)

Since the invention of pacemaker implementation, the direct capture and pacing of conduction systems have consistently drawn the attention of specialists. Scherlag *et al*.^21^ first described the method for pacing in animal studies. However, permanent HBP in patients was first demonstrated in the year 2000 by Deshmukh *et al*.,^22^ which has emerged as a significant advancement in pacing via the conduction system. This gap in the development of HBP and its utilization can be explained by technical challenges with placing pacing leads. Later development of delivery systems, pacing leads, and mapping catheters and a better understanding of cardiac anatomy and physiology led to new interest in HBP. HBP has demonstrated a lower all-cause mortality, reduced heart failure hospitalizations, and a less likely upgrade to cardiac resynchronization therapy (CRT) in comparison with RVP.^23^ HBP has also been associated with elevated LVEF and improved mechanical synchrony in a randomized clinical trial (RCT).^24^ However, HBP faces challenges, including the risk of lead displacement, anatomical complexities due to disease or other procedures, as well as increases in capture threshold and loss of capture during short- and long-term follow-ups, necessitating more frequent lead revisions.^25,26^ LBBAP has emerged as an alternative conduction system pacing method. It has gained greater acceptance since its first description in clinical practice in 2017 by Huang *et al*.^27^ This approach has exhibited stable pacing thresholds and preserved LV synchrony in patients with traditional pacemaker indications following bradycardia.^28,29^ Additionally, LBBAP has been proposed as an alternative method to BVP for patients with heart failure with reduced LVEF combined with either a wide or narrow QRS width, demonstrating improved LV systolic function in several randomized and observational studies.^30^ A major advantage of the method is that the pacing site is distal to the His bundle and proximal LBBB, which are the most pathological and vulnerable parts of the conduction system.^31^ LBBAP implantation is considered faster and less complex than HBP because of the widespread intraventricular septal localization of left bundle branch fascicules. Moreover, LBBAP has been shown to have a higher success rate, better sensing, and a lower capture threshold compared with HBP.^32^ LBBAP has also been found to be a feasible method with good and stable pacing parameters in CRT non-responder patients. In these patients, LBBAP resulted in significant clinical improvements and echocardiographic remodelling, contributing to better overall clinical outcomes.^33^ Moreover, LBBAP was found to lower the incidence of sustained and non-sustained ventricular tachyarrhythmias and new onset of atrial fibrillation in comparison with BVP.^9^

There are some clear advantages from an implanter’s point of view in favour of LBBAP. Lower pacing thresholds and better sensing are frequently observed compared with HBP, and the procedure is generally regarded as easier to perform by most implanting physicians. Importantly, HBP has exhibited a vulnerability with frequent incidents of late pacing threshold increase. The fear of loss of capture has often led to the implantation of a second ‘back-up lead’ in RV apex. This phenomenon does not appear to be as prevalent with LBBAP. Additionally, there is speculation about a higher likelihood of pacing from a site below an intrinsic block with LBBAP. These differences have positioned LBBAP as the preferred technique within conduction system pacing, and HBP is now being largely abandoned.

Furthermore, conduction system pacing has emerged as a viable alternative to BVP in patients requiring CRT, particularly demonstrating superiority in cases of LBBB. This approach not only leads to shortened QRS complexes but also enhances both systolic and diastolic function. The lack of acute response parameters has been the Achilles heel of CRT for over two decades, and several methods have been proposed to guide the LV lead into an optimal position. However, their accuracy may be compromised in patients with myocardial fibrosis, leading to suboptimal outcomes. In contrast, intraprocedural measurements for confirming left bundle branch capture offer better accuracy and can aid the decision to employ alternative methods such as conventional CRT or a combination of LBBAP and BVP known as left bundle branch optimized CRT (LOT-CRT). This technique has been shown to be feasible and safe for CRT.^34^ By utilizing the expertise of high-volume centres and skilled personnel, LOT-CRT enables swift conversion to CRT without delay. Similarly, an unsuccessful LBBAP will immediately be acknowledged and abandoned in favour of BVP. These might be potential arguments for introducing LBBAP as a first-line therapy in patients with classic indications for CRT.

However, it is important to understand that conduction system pacing has limitations and areas of uncertainty, leading to a knowledge gap. One notable limitation is the scarcity of long-term prognostic data beyond 12 months, particularly from RCTs, which are essential for establishing the efficacy and safety profiles of these pacing modalities. Moreover, while conduction system pacing offers promising alternatives, it presents procedural challenges. For example, achieving stable lead fixation with acceptable lead parameters can be challenging in HBP procedures. LBBAP procedures carry a risk of septal perforation with reported incidence rates between 0.3 and 13.9% in various studies.^35,36^ The concern with the late pacing threshold increase in HBP clearly illustrates the need for long-term data when new methods are introduced.^26^ In terms of cardiac electro-mechanics, LBBAP has been associated with delayed RV activation and a tendency towards an incomplete right bundle branch block pattern. However, the long-term effects of this delay are not well studied, as indicated by available literature.^37^

Moreover, limitations in lead placement in patients with septal fibrosis and enlarged cardiac chambers can also pose additional challenges for LBBAP implantations.^38^ Notably, a lower scar burden has been identified as a strong predictor of LBBAP response, and pre-procedure cardiac magnetic resonance scar evaluation is proposed as an informative tool for assessing LBBAP CRT responders.^39^ Additionally, intracardiac echocardiography–guided implantation during proximal LBBAP has been proposed to reduce procedure time and improve success rates.^40^

## Guidelines recommendations

The significance of conduction system pacing has been acknowledged in both European and American pacing guidelines.^41,42^ However, the 2018 American College of Cardiology/American Heart Association/Heart Rhythm Society Guideline contained only one recommendation pertaining to conduction system pacing. It stipulated that HBP and CRT were preferred over RVP in patients requiring permanent pacing, with an LVEF ranging from 36 to 50%, as a preventative measure against heart failure. Since that time, additional data have been acquired regarding conduction system pacing in patients requiring a conventional pacemaker or CRT. This culminated in a guideline on cardiac physiologic pacing for the avoidance and mitigation of heart failure by the Heart Rhythm Society, Asia Pacific Heart Rhythm Society, and Latin American Heart Rhythm Society.^43^ This guideline predominantly employed the term ‘cardiac physiologic pacing’, encompassing both conduction system pacing and biventricular pacing. Notably, this guideline provided multiple recommendations favouring conduction system pacing, including the use of physiologic pacing for patients with normal LVEF but a high projected burden of ventricular pacing. LBBAP was also recommended as an alternative to RVP for patients with normal LVEF and an anticipated low burden of ventricular pacing. In patients with sinus rhythm, LVEF ≤35%, LBBB with a QRS duration ≥150 ms, and New York Heart Association (NYHA) Classes II–IV symptoms, where BVP was unsuccessful, conduction system pacing (HBP with LBBB correction or LBBAP) was deemed reasonable. Notably, the guideline introduced the notion of revising cardiac implantable electronic devices to conduction system pacing for patients with pacemaker-induced cardiomyopathy.

The most recent European pacing guideline, published in 2021, recommended HBP as an alternative to BVP in patients in whom coronary sinus lead implantation is unsuccessful.^41^ Additionally, HBP was recommended as an alternative to RVP for patients with atrioventricular block and LVEF >40%, according to the 2021 European Society of Cardiology guidelines. However, all guidelines acknowledged the need for further studies to better characterize the clinical benefits of HBP over RVP.

## Conduction system pacing and RVP: comparative studies

Studies comparing conduction system pacing and RVP have focused on patients requiring pacemaker implantation following atrioventricular nodal ablation and on patients with standard bradycardia indication.

Although HBP was first described in the 1960s, its first clinical use was reported in the year 2000.^21,22^ HBP has been studied extensively for its effects on myocardial function. In one of the earliest interpatient acute comparison studies, Catanzariti *et al*.^8^ demonstrated that HBP could prevent LV dyssynchrony, mitigating outcomes such as mitral regurgitation and LV global function deterioration observed with RVP. Other studies examined the feasibility, safety, and effects of permanent para-Hisian pacing.^44^ While no significant differences in LVEF were observed between HBP and RVP groups during follow-up, reductions in mitral and tricuspid regurgitation were noteworthy, potentially mediated by less interventricular dyssynchrony demonstrated through QRS shortening with HBP. Zanon *et al*.^45^ found less myocardial dyssynchrony in patients with HBP compared with RVP by tissue Doppler imaging. They did not find any differences in LVEF and LV dimensions in both groups during follow-up, whereas mitral regurgitation was higher in RVP. Another crossover study conducted by Kronborg *et al*.^24^ aimed to evaluate the long-term effects of HBP in LV function compared with RVP in consecutive patients. They found that HBP preserves LVEF compared with RVP, but there were no other clinical improvements. They also found shorter differences in time to peak systolic velocity between basal segments in patients with HBP. Another interesting point was that HBP preserved LVEF and had benefits for patients with deteriorated LVEF during RVP or hypertension. Similarly, Vijayaraman *et al*.^26^ reported LVEF preservation and less tricuspid regurgitation in HBP in their retrospective case–control study. Furthermore, paced QRS was significantly narrower in the HBP group and remained unchanged during follow-up.

The focus on LBBAP has grown in recent years. Researchers have demonstrated its feasibility and superiority in terms of LV mechanical synchrony over RVP.^27^ Hou *et al*.^46^ compared LBBAP, HBP, and RVP through a phase analysis of single-photon-emission computed tomography myocardial perfusion imaging and demonstrated that the LV mechanical synchrony of LBBAP patients was similar to that of HBP patients and better than that of RVP patients. Likewise, Cai *et al*.^47^ reported LBBAP-induced LV mechanical synchrony similar to native conduction and superior RVP. Paced QRS in LBBAP was wider than native conduction due to the RV activation delay, but narrower than RVP. Moreover, the systolic dyssynchrony index in the LBBAP group was not statistically different from native conduction, whereas it was significantly higher in the RVP group. The standard deviation of time-to-peak contraction velocity in LV 12 segments also showed the same results. Das *et al*.^48^ also studied myocardial synchrony and LVEF in LBBAP compared with RVP. They found that LBBAP patients had a higher LVEF, a lower LV internal dimension in diastole, and decreased interventricular delay compared with RVP recipients. Additionally, intraventricular delay, as calculated by septal to posterior motion delay, was less pronounced in the LBBAP group. Similarly, Mao *et al*. demonstrated better myocardial synchrony in patients with LBBAP compared with those who received RVP. This was demonstrated by the LV global longitudinal strain lateral wall to septal wall work difference and global and lateral myocardial work.^49^ Notably, the RVP group demonstrated a significantly impaired septal work relative to lateral wall work, resulting in large LW–SW work differences. In contrast, the LBBAP group demonstrated a consistent and stable septal work, leading to significantly lower lateral wall-septal wall differences. Importantly, this difference in the LBBAP group was not significantly increased during follow-up, indicating the enduring efficacy of LBBAP in maintaining myocardial synchrony over time. Tricuspid regurgitation was significantly more prevalent in the RVP group than in the LBBAP group after a 1-year follow-up period. Ponnusamy and Vijayaraman compared LBBAP, HBP, and RVP in a two-centre retrospective cohort study and found that LVEF improvement was the same in both LBBAP and HBP, whereas RVP caused a significant reduction in LVEF. They also found that LBBAP and HBP caused a significant shortening of QRS compared with RVP. However, these changes were not significantly different in both groups (LBBAP and HBP).^50^

In summary, compared with RVP, HBP has been associated with preserved LVEF, less tricuspid and mitral regurgitations, and shorter QRS in patients with pacemaker indication. Similarly, LBBAP has also shown better LV mechanical synchrony and a higher LVEF compared with RVP, as evidenced by a lower systolic dyssynchrony index and inter- and intraventricular delays in these patients.

*Table 1* summarizes studies comparing LV function in conduction system pacing and RVP.

**Table 1:** Summary of studies comparing conduction system pacing and RVP with echocardiographic findings

Authors	Study type	No. of patients	Compared types	Pacing indication	Follow-up	Outcomes	ΔLVEF	LVEF
Catanzariti *et al*.^8^	Prospective, intrapatient acute comparison	23	RVP, HBP and mHBP	SND, AVB, or AF with slow rate		IVD, SPD, and TDId		
Occhetta *et al*.^44^	Crossover, patient blind, randomized	18	RVP and HBP	AVN ablation for AF	6 months	EF, IVD, MR, and TR		52% baseline to 50% RVP and 53.4% HBP (*P* = ns)
Zanon *et al*.^45^	Prospective, crossover mid-term	12	RVP and HBP	AVB or AF with slow rate	3 months	LVEF, myocardial dyssynchrony		59.8% baseline to 61% RVP and 63% HBP (*P* > 0.05)
Kronborg *et al*.^24^	Prospective, randomized, single-centre crossover	34	RVP and HBP	AVB	12 months	LVEF, time-to-peak systolic velocity		50% RVP and 55% HBP (*P* = 0.005)
Vijayaraman *et al*.^26^	Observational, retrospective, case–control	173	RVP and HBP	Bradycardia	5 years	LVEF		57% baseline to 52% (RVP; *P* = 0.002) and 55% (HBP; *P* = 0.13)
Das *et al*.^48^	Prospective, single centre	50	RVP and LBBAP	Bradycardia	6 months	LVEF, LVEDd, IVD		60% RVP and 64% LBBAP (*P* = 0.013)
Mao *et al*.^49^	Observational, two-centre	82	RVP and LBBAP	Bradycardia	1 year	ΔLVEF, LVGLS	−7.4 and 0.3% (*P* < 0.01)	58% RVP and 65% LBBAP (*P* < 0.01)
Ponnusamy *et al*.^50^	Retrospective, two-centre	121	RVP, HBP, and LBBAP	Bradycardia	4 weeks	ΔLVEF	−4%, + 5%, + 4 (RVP, HBP, LBBAP, *P* = 0.001)	

AF, atrial fibrillation; AVB, atrioventricular blockage; AVN, atrioventricular node; CSP, conduction system pacing; EF, ejection fraction; HBP, His bundle pacing; IVD, interventricular dimension; LBBAP, left bundle branch area pacing; LVEDd, left ventricular end-diastolic dimension; LVEF, left ventricular ejection fraction; LVGLS, left ventricular global longitudinal strain; mHBP, modified His bundle pacing (His bundle pacing + right ventricular pacing); MR, mitral regurgitation; RVP, right ventricular pacing; SND, sinus node dysfunction; SPD, septal to left posterior wall motion delay; TDId, maximal difference between tissue Doppler imaging systolic velocities of basal left ventricular segment; TR, tricudpid regurgitation.

## Conduction system pacing and biventricular pacing: comparative studies in CRT indication

BVP is a cornerstone pacing technique for patients requiring cardiac CRT. However, conduction system pacing is gaining traction in this patient cohort. Numerous studies have demonstrated comparatively substantial improvements in ventricular ejection fraction for individuals undergoing conduction system pacing vs. BVP. Lustgarten *et al*.^51^ conducted a study illustrating that HBP can normalize QRS intervals in patients with bundle branch block and CRT indications, yielding a 6-month CRT response equivalent to that achieved by BVP. While no significant LVEF differences were found between patients with BVP and HBP, QRS duration was relatively shortened in selective HBP recipients.^30^ Wu *et al*.^52^ compared the clinical outcomes of HBP, LBBAP, and BVP in patients with typical LBBB requiring CRT. Both HBP and LBBAP elicited structural and functional LV improvements compared with BVP. Effective resynchronization was evident in both conduction system pacing methods, as evidenced by reduced QRS duration and improved LVEF, surpassing the effectiveness of BVP.

The His-SYNC pilot trial did not show significant electrocardiographic or echocardiographic improvements in the HBP group compared with BVP.^53^ The notable crossover rate could, however, have influenced their results. In contrast, Li *et al*.^54^ analysed LBBAP in patients with CRT indications, uncovering that LBBAP significantly narrowed QRS intervals, improved LVEF, and marginally reduced LVEDd relative to BVP. Similarly, Vinther *et al*.,^55^ in a randomized clinical study with the same inclusion criteria, found comparable echocardiographic parameters, clinical symptoms, and physical capabilities between HBP and BVP groups. Kato *et al*.^56^ explored acute haemodynamic effects, echocardiographic features, and clinical outcomes of HBP and BVP in patients with heart failure and LBBB over a 1-year follow-up period. The study revealed that HBP improved LV relaxation, while BVP did not. Both methods brought about clinical and echocardiographic enhancements, such as reductions in left atrial diameter, mitral regurgitation, LV end-systolic volume, and improvement of LVEF, which were more prominent and rapid in the HBP group. All HBP emerged as CRT super-responders, contrasting with 60% of the BVP group. Vijayaraman *et al*.^57^ observed greater LVEF improvement in the conduction system pacing group compared with the BVP group, with a more pronounced effect seen in patients with LBBB. Chen *et al*.^58^ expanded on these findings, analysing more echocardiographic data over a 1-year follow-up. While the response rate (improvement of LVEF >5%) remained consistent in the LBBAP and BVP groups after a year, the improvement in LVEF and reduction of LVEDd were greater in patients who underwent LBBAP. Ezzeddine *et al*. extended their study to encompass not only patients with CRT indication, but also those requiring a permanent pacemaker. Their findings indicated that conduction system pacing recipients (both HBP and LBBAP) exhibited a higher CRT response, characterized by an LVEF increase of ≥5% 6 months post-implantation, when compared with BVP recipients.^59^ In the I-CLAS study, which is one of the latest retrospective studies with 1778 patients from 15 international centres, it was found that LBBAP produced significantly higher rates of echocardiographic response and hyper-response compared with BVP in all patients and in patients with LBBB.^60^ Interestingly, in this study, echocardiographic response rates (ΔLVEF ≥5%) were found to be significantly greater in the LBBAP group than in the BVP group (81.7 vs. 68.2%; *P* < 0.001).

In the patient group with CRT indication, conduction system pacing has shown superior LV mechanical function characterized by a higher LVEF and narrower QRS. Conduction system pacings have also been found to improve LV diastolic function compared with BVP. Furthermore, the recipients of conduction system pacing showed a better response than patients undergoing BVP.

*Table 2* summarizes studies comparing LV function in conduction system pacing BVP.

**Table 2:** Summary of studies comparing conduction system pacing and BVP with echocardiographic findings

Authors	Study type	No. of patients	Compared types	Follow-up	Outcomes	ΔLVEF	LVEF	NYHA class improvement
Lustgarten *et al*.^51^	Crossover, RCT	29	BVP, HBP	6 months	LVEF		26% (baseline) to 32% (HBP; *P* = 0.043) and 31% (BVP; *P* = 0.02)	2.9 (baseline) to 1.9 (BVP; *P* < 0.001) and 1.9 (HBP; *P* < 0.001)
Wu *et al*.^52^	Prospective, non-RCT	137	BVP, HBP, LBBAP	12 months	LVEF	16.7% (BVP), 23.9% (HBP), and 24.0% (LBBAP)		2.8 (baseline) to 1.9 (BVP)
Upadhyay *et al*.^53^	Pilot trial of His-SYNC study (prospective, RCT)	40	BVP, HBP	6 months	LVEF	5.9% (BVP) and 7.2% (HBP) (*P* = 0.17)	28% (baseline) to 34.6% (HBP; *P* < 0.001) and 28% (baseline) to 32% (BVP; *P* < 0.001)	
Li *et al*.^54^	Prospective, observational, multicentre study	81	BVP, LBBAP	6 months	LVEF LVEDd	7% (BVP) and 17.1% (LBBAP) (*P* < 0.001)	27–35% (BVP) and 29–44% (LBBAP)	3.0 (baseline) to 2.3 (BVP) and 3.1 (baseline) to 1.5 (LBBAP)
Vinther *et al*.^55^	RCT	50	BVP, HBP	6 months	LVEF, LVESV	13% (BVP) and 17% (HBP) (*P* = 0.053)		2.4 (baseline) to 1.9 (BVP; *P* < 0.001) and 1.8 (HBP; *P* < 0.001)
Kato *et al*.^56^	Non-RCT, single centre	14	BVP, HBP	12 months	LVEF, LVESV	14.8% (BVP) and 29.6% (HBP) (*P* < 0.05)		2.6–1.2 (BVP; *P* < 0.05) and 2.7–1.2 (HBP; *P* < 0.001)
Vijayaraman *et al*.^57^	Retrospective, observational study	477	BVP, CSP (HBP and LBBAP)	27 months	LVEF	7 and 13.6% (*P* < 0.001)	39.7% CSP and 33.1% BVP (*P* < 0.001)	
Chen *et al*.^58^	Non-randomized, prospective, multicentre, observational study	100	LBBAP, BVP	12 months	LVEF, LVEDD, LVEDV	21 and 15% (*P* < 0.05)	29–49 and 28–43% (*P* < 0.001)	
Ezzeddine *et al*.^59^	Retrospective cohort study	238	BVP, HBP, LBBAP	6 months	LVEF, LVESV	7.3% (BVP) and 10.4% (CSP) (*P* = 0.042)		
Vijayaraman *et al*.^60^	Multicentre, international, observational, retrospective comparative study	1778	BVP, LBBAP	6 months	LVEF, LVEDd	10% (BVP) vs. 13% (LBBAP) (*P* < 0.001)	From 26.4 to 37.3% (BVP, *P* < 0.001); from 26.1 to 41.4% (LBBAP, *P* < 0.001)	From 2.69 to 2.19 (BVP, *P* < 0.001); from 2.82 to 2.01 (LBBAP, *P* < 0.001)

BVP, biventricular pacing; CSP, conduction system pacing; HBP, high bundle pacing; LBBAP, left bundle branch area pacing; LVEDd, left ventricular end-diastolic dimension; LVEDV, left ventricular end-diastolic volume; LVEF, left ventricular ejection fraction; LVESV, left ventricular end-systolic volume; NYHA, New York Heart Association; RCT, randomized clinical trial.

## Advancements in conduction system pacing and future perspectives

In this comprehensive review, we have examined the existing literature concerning conduction system pacing in contrast to conventional RVP and BVP, with a particular emphasis on myocardial function. The analysis reveals that conduction system pacing, encompassing HBP and LBBAP, might herald a new era of pacing strategies that offer a more synchronous myocardial function compared with RVP. Through its physiological approach, conduction system pacing demonstrates important advantages over traditional pacing methods. This is evidenced by a narrower QRS, preserved LVEF, and reduced rates of mitral and tricuspid regurgitation over extended periods. However, no direct, large-scale comparison of the two different conduction system pacing methods exists.

As conduction system pacing has gained increased attention in recent years, ongoing clinical randomized trials will probably answer critical questions regarding its safety and efficacy. However, more studies focusing on myocardial mechanics in patients with such a condition are needed to understand the benefits and limitations of this pacing approach. Particularly, studies of the mechanical and clinical features of delayed RV activation in LBBAP over extended periods will be essential for clinical decision-making. For example, the ‘pace and ablate’ strategy for patients with atrial fibrillation will appear much more attractive if long-term results with conduction system pacing indicate a reduction in pacing-induced cardiomyopathy. Additionally, exploring hybrid approaches of conduction system pacing, such as His optimized CRT and left bundle branch optimized CRT, warrants attention to assess their impact on myocardial work and clinical outcome. Future studies such as these will not only enhance our understanding of conduction system pacing but also offer insights into refining pacing strategies to optimize patient outcomes.

## Summary

LBBAP is a promising method in cardiac pacing. In patients with bradycardia and narrow QRS complexes, the goal of pacing is to cause as little harm as possible. In contrast, the goal of pacing in heart failure patients with LBBB is to improve electro-mechanic cardiac function. LBBAP might be the solution for the majority of these patient groups. However, as the field of conduction system pacing continues to evolve, further investigations are warranted. Long-term RCTs and trials focusing on myocardial mechanics are pivotal for a comprehensive understanding of the clinical benefits and long-term effects of innovative pacing methods such as the ones mentioned in this article.

## Data Availability

No new data were generated or analysed in support of this research.

## References

[1] Tse H-F , Lau C-P. Long-term effect of right ventricular pacing on myocardial perfusion and function. J Am Coll Cardiol 1997;29:744–9. 9091519 10.1016/s0735-1097(96)00586-4

[2] Tops LF , Schalij MJ, Bax JJ. The effects of right ventricular apical pacing on ventricular function and dyssynchrony. J Am Coll Cardiol 2009;54:764–76. 19695453 10.1016/j.jacc.2009.06.006

[3] Bristow MR , Saxon LA, Boehmer J, Krueger S, Kass DA, De Marco T et al Cardiac-resynchronization therapy with or without an implantable defibrillator in advanced chronic heart failure. N Engl J Med 2004;350:2140–50. 15152059 10.1056/NEJMoa032423

[4] Khurshid S , Obeng-Gyimah E, Supple GE, Schaller R, Lin D, Owens AT et al Reversal of pacing-induced cardiomyopathy following cardiac resynchronization therapy. JACC Clin Electrophysiol 2018;4:168–77. 29749933 10.1016/j.jacep.2017.10.002

[5] Ploux S , Eschalier R, Whinnett ZI, Lumens J, Derval N, Sacher F et al Electrical dyssynchrony induced by biventricular pacing: implications for patient selection and therapy improvement. Heart Rhythm 2015;12:782–91. 25546811 10.1016/j.hrthm.2014.12.031

[6] Daubert JC , Ritter P, Breton H, Gras D, Leclercq C, Lazarus A et al Permanent left ventricular pacing with transvenous leads inserted into the coronary veins. Pacing Clin Electrophysiol 1998;21:239–45. 9474680 10.1111/j.1540-8159.1998.tb01096.x

[7] Prinzen FW , Vernooy K, Auricchio A. Cardiac resynchronization therapy. Circulation 2013;128:2407–18. 24276876 10.1161/CIRCULATIONAHA.112.000112

[8] Catanzariti D , Maines M, Cemin C, Broso G, Marotta T, Vergara G. Permanent direct His bundle pacing does not induce ventricular dyssynchrony unlike conventional right ventricular apical pacing. J Interv Card Electrophysiol 2006;16:81–92. 17115267 10.1007/s10840-006-9033-5

[9] Herweg B , Sharma PS, Cano O, Ponnusamy SS, Zanon F, Jastrzebski M et al Arrhythmic risk in biventricular pacing compared with left bundle branch area pacing: results from the I-CLAS study. Circulation 2024;149:379–90. 37950738 10.1161/CIRCULATIONAHA.123.067465

[10] Prinzen FW , Hunter WC, Wyman BT, McVeigh ER. Mapping of regional myocardial strain and work during ventricular pacing: experimental study using magnetic resonance imaging tagging. J Am Coll Cardiol 1999;33:1735–42. 10334450 10.1016/s0735-1097(99)00068-6 PMC2041911

[11] Kass DA . An epidemic of dyssynchrony. J Am Coll Cardiol 2008;51:12–7. 18174030 10.1016/j.jacc.2007.09.027

[12] Delgado V , Tops LF, Trines SA, Zeppenfeld K, Ajmone Marsan N, Bertini M et al Acute effects of right ventricular apical pacing on left ventricular synchrony and mechanics. Circ Arrhythm Electrophysiol 2009;2:135–45. 19808458 10.1161/CIRCEP.108.814608

[13] Sarvari SI , Sitges M, Sanz M, Viu JMT, Edvardsen T, Stokke TM et al Left ventricular dysfunction is related to the presence and extent of a septal flash in patients with right ventricular pacing. Europace 2017;19:289–96. 28175277 10.1093/europace/euw020

[14] Kaye GC , Linker NJ, Marwick TH, Pollock L, Graham L, Pouliot E et al Effect of right ventricular pacing lead site on left ventricular function in patients with high-grade atrioventricular block: results of the protect-pace study. Eur Heart J 2015;36:856–62. 25189602 10.1093/eurheartj/ehu304

[15] Galand V , Martins RP, Donal E, Behar N, Crocq C, Soulié GG et al Septal versus apical pacing sites in permanent right ventricular pacing: the multicentre prospective SEPTAL-PM study. Arch Cardiovasc Dis 2022;115:288–94. 35221255 10.1016/j.acvd.2021.12.007

[16] Mariani MV , Piro A, Forleo GB, Della RD, Natale A, Miraldi F et al Clinical, procedural and lead outcomes associated with different pacing techniques: a network meta-analysis. Int J Cardiol 2023;377:52–9. 36736670 10.1016/j.ijcard.2023.01.081

[17] Prinzen FW , Lumens J, Duchenn J, Vernooy K. Electro-energetics of biventricular, septal and conduction system pacing. Arrhythm Electrophysiol Rev 2021;10:250–7. 35106177 10.15420/aer.2021.30 PMC8785089

[18] Meiburg R , Rijks JHJ, Beela AS, Bressi E, Grieco D, Delhaas T et al Comparison of novel ventricular pacing strategies using an electro-mechanical simulation platform. Europace 2023;25:euad144. 37306315 10.1093/europace/euad144 PMC10259067

[19] Hua J , Wang C, Kong Q, Zhang Y, Wang Q, Xiong Z et al Comparative effects of left bundle branch area pacing, His bundle pacing, biventricular pacing in patients requiring cardiac resynchronization therapy: a network meta-analysis. Clin Cardiol 2022;45:214–23. 35128691 10.1002/clc.23784 PMC8860481

[20] Gin J , Chow CL, Voskoboinik A, Nalliah C, Wong C, Van Gaal W et al Improved outcomes of conduction system pacing in heart failure with reduced ejection fraction—a systematic review and meta-analysis. Heart Rhythm 2023;20:1178–87. 37172670 10.1016/j.hrthm.2023.05.010

[21] Scherlag BJ , Kosowsky BD, Damato AN. A technique for ventricular pacing from the His bundle of the intact heart. J Appl Physiol 1967;22:584–7. 6020246 10.1152/jappl.1967.22.3.584

[22] Deshmukh P , Casavant DA, Romanyshyn M, Anderson K. Permanent, direct His-bundle pacing. Circulation 2000;101:869–77. 10694526 10.1161/01.cir.101.8.869

[23] Abdelrahman M , Subzposh FA, Beer D, Durr B, Naperkowski A, Sun H et al Clinical outcomes of His bundle pacing compared to right ventricular pacing. J Am Coll Cardiol 2018;71:2319–30. 29535066 10.1016/j.jacc.2018.02.048

[24] Kronborg MB , Mortensen PT, Poulsen SH, Gerdes JC, Jensen HK, Nielsen JC. His or para-His pacing preserves left ventricular function in atrioventricular block: a double-blind, randomized, crossover study. Europace 2014;16:1189–96. 24509688 10.1093/europace/euu011

[25] Teigeler T , Kolominsky J, Vo C, Shepard RK, Kalahasty G, Kron J et al Intermediate-term performance and safety of His-bundle pacing leads: a single-center experience. Heart Rhythm 2021;18:743–9. 33418127 10.1016/j.hrthm.2020.12.031

[26] Vijayaraman P , Naperkowski A, Subzposh FA, Abdelrahman M, Sharma PS, Oren JW et al Permanent His-bundle pacing: long-term lead performance and clinical outcomes. Heart Rhythm 2018;15:696–702. 29274474 10.1016/j.hrthm.2017.12.022

[27] Huang W , Su L, Wu S, Xu L, Xiao F, Zhou X et al A novel pacing strategy with low and stable output: pacing the left bundle branch immediately beyond the conduction block. Can J Cardiol 2017;33:1736.e1–e3. 10.1016/j.cjca.2017.09.013 29173611

[28] Li X , Li H, Ma W, Ning X, Liang E, Pang K et al Permanent left bundle branch area pacing for atrioventricular block: feasibility, safety, and acute effect. Heart Rhythm 2019;16:1766–73. 31048065 10.1016/j.hrthm.2019.04.043

[29] Padala SK , Master VM, Terricabras M, Chiocchini A, Garg A, Kron J et al Initial experience, safety, and feasibility of left bundle branch area pacing. JACC Clin Electrophysiol 2020;6:1773–82. 33357573 10.1016/j.jacep.2020.07.004

[30] Vijayaraman P , Ponnusamy S, Cano Ó, Sharma PS, Naperkowski A, Subsposh FA et al Left bundle branch area pacing for cardiac resynchronization therapy. JACC Clin Electrophysiol 2021;7:135–47. 33602393 10.1016/j.jacep.2020.08.015

[31] Zhang S , Zhou X, Gold MR. Left bundle branch pacing: JACC review topic of the week. J Am Coll Cardiol 2019;74:3039–49. 31865972 10.1016/j.jacc.2019.10.039

[32] Zhuo W , Zhong X, Liu H, Yu J, Chen Q, Hu J et al Pacing characteristics of His bundle pacing vs. Left bundle branch pacing: a systematic review and meta-analysis. Front Cardiovasc Med 2022;9:849143. 35391846 10.3389/fcvm.2022.849143 PMC8980919

[33] Chen X , Jin Q, Qiu Z, Qian C, Liang Y, Wang J et al Outcomes of upgrading to LBBP in CRT nonresponders: a prospective, multicenter, nonrandomized, case-control study. JACC Clin Electrophysiol 2024;10:108–20. 37943191 10.1016/j.jacep.2023.08.031

[34] Jastrzębski M , Moskal P, Huybrechts W, Curila K, Sreekumar P, Rademakers LM et al Left bundle branch–optimized cardiac resynchronization therapy (LOT-CRT): results from an international LBBAP collaborative study group. Heart Rhythm 2022;19:13–21. 34339851 10.1016/j.hrthm.2021.07.057

[35] Ponnusamy SS , Basil W, Vijayaraman P. Electrophysiological characteristics of septal perforation during left bundle branch pacing. Heart Rhythm 2022;19:728–34. 35066178 10.1016/j.hrthm.2022.01.018

[36] Vijayaraman P , Subzposh FA, Naperkowski A, Panikkath R, John K, Mascarenhas V et al Prospective evaluation of feasibility and electrophysiologic and echocardiographic characteristics of left bundle branch area pacing. Heart Rhythm 2019;16:1774–82. 31136869 10.1016/j.hrthm.2019.05.011

[37] Ali N , Arnold AD, Miyazawa AA, Keene D, Chow J-J, Little I et al Comparison of methods for delivering cardiac resynchronization therapy: an acute electrical and haemodynamic within-patient comparison of left bundle branch area, His bundle, and biventricular pacing. Europace 2023;25:1060–7. 36734205 10.1093/europace/euac245 PMC10062293

[38] Jastrzębski M , Kiełbasa G, Cano O, Curila K, Heckman L, De Pooter J et al Left bundle branch area pacing outcomes: the multicentre European MELOS study. Eur Heart J 2022;43:4161–73. 35979843 10.1093/eurheartj/ehac445 PMC9584750

[39] Chen Z , Ma X, Gao Y, Wu S, Xu N, Chen F et al Cardiac magnetic resonance–derived myocardial scar is associated with echocardiographic response and clinical prognosis of left bundle branch area pacing for cardiac resynchronization therapy. Europace 2023;25:euad326. 37926926 10.1093/europace/euad326 PMC10639094

[40] Kuang X , Zhang X, Cui Y, Wei F, Wu P, Gao X et al Intracardiac echocardiography–guided implantation for proximal left bundle branch pacing. Circ Arrhythm Electrophysiol 2023;16:e011408. 36924221 10.1161/CIRCEP.122.011408 PMC10108837

[41] Glikson M , Nielsen JC, Kronborg MB, Michowitz Y, Auricchio A, Barbash IM et al 2021 ESC guidelines on cardiac pacing and cardiac resynchronization therapy. Eur Heart J 2021;42:3427–520. 34586378 10.1093/eurheartj/ehab699

[42] Kusumoto FM , Schoenfeld MH, Barrett C, Edgerton JR, Ellenbogen KA, Gold MR et al 2018 ACC/AHA/HRS guideline on the evaluation and management of patients with bradycardia and cardiac conduction delay: a report of the American College of Cardiology/American Heart Association task force on clinical practice guidelines and the Heart Rhythm Society. Circulation 2019;140:e382–482. 30586772 10.1161/CIR.0000000000000628

[43] Chung MK , Patton KK, Lau C-P, Dal Forno ARJ, Al-Khatib SM, Arora V et al 2023 HRS/APHRS/LAHRS guideline on cardiac physiologic pacing for the avoidance and mitigation of heart failure. Heart Rhythm 2023;20:e17–91. 37283271 10.1016/j.hrthm.2023.03.1538 PMC11062890

[44] Occhetta E , Bortnik M, Magnani A, Francalacci G, Piccinino C, Plebani L et al Prevention of ventricular desynchronization by permanent para-Hisian pacing after atrioventricular node ablation in chronic atrial fibrillation. J Am Coll Cardiol 2006;47:1938–45. 16697308 10.1016/j.jacc.2006.01.056

[45] Zanon F , Bacchiega E, Rampin L, Aggio S, Baracca E, Pastore G et al Direct His bundle pacing preserves coronary perfusion compared with right ventricular apical pacing: a prospective, cross-over mid-term study. Europace 2008;10:580–7. 18407969 10.1093/europace/eun089

[46] Hou X , Qian Z, Wang Y, Qiu Y, Chen X, Jiang H et al Feasibility and cardiac synchrony of permanent left bundle branch pacing through the interventricular septum. Europace 2019;21:1694–702. 31322651 10.1093/europace/euz188

[47] Cai B , Huang X, Li L, Guo J, Chen S, Meng F et al Evaluation of cardiac synchrony in left bundle branch pacing: insights from echocardiographic research. J Cardiovasc Electrophysiol 2020;31:560–9. 31919928 10.1111/jce.14342 PMC7027438

[48] Das A , Islam SS, Pathak SK, Majumdar I, Sharwar SA, Saha R et al Left bundle branch area. A new site for physiological pacing: a pilot study. Heart Vessels 2020;35:1563–72. 32458055 10.1007/s00380-020-01623-y

[49] Mao Y , Duchenne J, Yang Y, Garweg C, Yang Y, Sheng X et al Left bundle branch pacing better preserves ventricular mechanical synchrony than right ventricular pacing: a two-center study. Eur Heart J Cardiovasc Imaging 2024;25:328–36. 37933672 10.1093/ehjci/jead296

[50] Ponnusamy SS , Vijayaraman P. Pacing for atrioventricular block with preserved left ventricular function: on-treatment comparison between His bundle, left bundle branch, and right ventricular pacing. Indian Pacing Electrophysiol J 2023;23:196–202. 37776973 10.1016/j.ipej.2023.09.006 PMC10685102

[51] Lustgarten DL , Crespo EM, Arkhipova-Jenkins I, Lobel R, Winget J, Koehler J et al His-bundle pacing versus biventricular pacing in cardiac resynchronization therapy patients: a crossover design comparison. Heart Rhythm 2015;12:1548–57. 25828601 10.1016/j.hrthm.2015.03.048

[52] Wu S , Su L, Vijayaraman P, Zheng R, Cai M, Xu L et al Left bundle branch pacing for cardiac resynchronization therapy: nonrandomized on-treatment comparison with His bundle pacing and biventricular pacing. Can J Cardiol 2021;37:319–28. 32387225 10.1016/j.cjca.2020.04.037

[53] Upadhyay GA , Vijayaraman P, Nayak HM, Verma N, Dandamudi G, Sharma PS et al On-treatment comparison between corrective His bundle pacing and biventricular pacing for cardiac resynchronization: a secondary analysis of the His-SYNC pilot trial. Heart Rhythm 2019;16:1797–807. 31096064 10.1016/j.hrthm.2019.05.009

[54] Li X , Qiu C, Xie R, Ma W, Wang Z, Li H et al Left bundle branch area pacing delivery of cardiac resynchronization therapy and comparison with biventricular pacing. ESC Heart Fail 2020;7:1711–22. 32400967 10.1002/ehf2.12731 PMC7373885

[55] Vinther M , Risum N, Svendsen JH, Møgelvang R, Philbert BT. A randomized trial of His pacing versus biventricular pacing in symptomatic HF patients with left bundle branch block (His-alternative). JACC Clin Electrophysiol 2021;7:1422–32. 34167929 10.1016/j.jacep.2021.04.003

[56] Kato H , Yanagisawa S, Sakurai T, Mizuno C, Ota R, Watanabe R et al Efficacy of His bundle pacing on LV relaxation and clinical improvement in HF and LBBB. JACC Clin Electrophysiol 2022;8:59–69. 34454880 10.1016/j.jacep.2021.06.011

[57] Vijayaraman P , Zalavadia D, Haseeb A, Dye C, Madan N, Skeete JR et al Clinical outcomes of conduction system pacing compared to biventricular pacing in patients requiring cardiac resynchronization therapy. Heart Rhythm 2022;19:1263–71. 35500791 10.1016/j.hrthm.2022.04.023

[58] Chen X , Ye Y, Wang Z, Jin Q, Qiu Z, Wang J et al Cardiac resynchronization therapy via left bundle branch pacing vs. optimized biventricular pacing with adaptive algorithm in heart failure with left bundle branch block: a prospective, multi-centre, observational study. Europace 2022;24:807–16. 34718539 10.1093/europace/euab249 PMC9071084

[59] Ezzeddine FM , Pistiolis SM, Pujol-Lopez M, Lavelle M, Wan EY, Patton KK et al Outcomes of conduction system pacing for cardiac resynchronization therapy in patients with heart failure: a multicenter experience. Heart Rhythm 2023;20:863–71. 36842610 10.1016/j.hrthm.2023.02.018 PMC10225322

[60] Vijayaraman P , Sharma PS, Cano Ó, Ponnusamy SS, Herweg B, Zanon F. Comparison of left bundle branch area pacing and biventricular pacing in candidates for resynchronization therapy. J Am Coll Cardiol 2023;82:228–41. 37220862 10.1016/j.jacc.2023.05.006

